# Supplementary material for: Unveiling the Bioactive Potential of the Invasive Jellyfish Phyllorhiza punctata Through Integrative Transcriptomic and Proteomic Analyses
Source: Biomolecules. 2025 Aug 4;15(8):1121. doi: 10.3390/biom15081121 (PMC12383608; doi:10.3390/biom15081121)
Supplement: Supplementary file 1 [file biomolecules-15-01121-s001.zip › Suplementary Material/FileS3/PEPFOLD4/XP_065053952_4606/clusters.html]

 PEP-FOLD clusters


Download archive of all models  here   

|  |  |  |
| --- | --- | --- |
| # | file | sOPEP |
| # cluster 1 - Size: 2 |  |  |
|  | XP\_065053952\_4606-model1 | -15.9831 |
|  | XP\_065053952\_4606-model1.1 | -15.8611 |
| # cluster 2 - Size: 1 |  |  |
|  | XP\_065053952\_4606-model2 | -8.90114 |
| # cluster 3 - Size: 17 |  |  |
|  | XP\_065053952\_4606-model3 | -8.64713 |
|  | XP\_065053952\_4606-model3.1 | -7.92964 |
|  | XP\_065053952\_4606-model3.2 | -7.63157 |
|  | XP\_065053952\_4606-model3.3 | -7.45172 |
|  | XP\_065053952\_4606-model3.4 | -7.43062 |
|  | XP\_065053952\_4606-model3.5 | -7.37363 |
|  | XP\_065053952\_4606-model3.6 | -7.33819 |
|  | XP\_065053952\_4606-model3.7 | -7.15315 |
|  | XP\_065053952\_4606-model3.8 | -7.14577 |
|  | XP\_065053952\_4606-model3.9 | -7.11346 |
|  | XP\_065053952\_4606-model3.10 | -6.94899 |
|  | XP\_065053952\_4606-model3.11 | -6.85103 |
|  | XP\_065053952\_4606-model3.12 | -6.7627 |
|  | XP\_065053952\_4606-model3.13 | -6.71997 |
|  | XP\_065053952\_4606-model3.14 | -6.71738 |
|  | XP\_065053952\_4606-model3.15 | -6.63787 |
|  | XP\_065053952\_4606-model3.16 | -6.62111 |
| # cluster 4 - Size: 2 |  |  |
|  | XP\_065053952\_4606-model4 | -8.46698 |
|  | XP\_065053952\_4606-model4.1 | -7.72707 |
| # cluster 5 - Size: 2 |  |  |
|  | XP\_065053952\_4606-model5 | -8.0848 |
|  | XP\_065053952\_4606-model5.1 | -7.51224 |
| # cluster 6 - Size: 1 |  |  |
|  | XP\_065053952\_4606-model6 | -7.90085 |
| # cluster 7 - Size: 3 |  |  |
|  | XP\_065053952\_4606-model7 | -7.90053 |
|  | XP\_065053952\_4606-model7.1 | -7.04504 |
|  | XP\_065053952\_4606-model7.2 | -6.99038 |
| # cluster 8 - Size: 1 |  |  |
|  | XP\_065053952\_4606-model8 | -7.76935 |
| # cluster 9 - Size: 4 |  |  |
|  | XP\_065053952\_4606-model9 | -7.76097 |
|  | XP\_065053952\_4606-model9.1 | -7.02148 |
|  | XP\_065053952\_4606-model9.2 | -6.90358 |
|  | XP\_065053952\_4606-model9.3 | -6.70799 |
| # cluster 10 - Size: 6 |  |  |
|  | XP\_065053952\_4606-model10 | -7.68946 |
|  | XP\_065053952\_4606-model10.1 | -7.56041 |
|  | XP\_065053952\_4606-model10.2 | -7.37174 |
|  | XP\_065053952\_4606-model10.3 | -7.14801 |
|  | XP\_065053952\_4606-model10.4 | -6.87497 |
|  | XP\_065053952\_4606-model10.5 | -6.56508 |
| # cluster 11 - Size: 1 |  |  |
|  | XP\_065053952\_4606-model11 | -7.61162 |
| # cluster 12 - Size: 2 |  |  |
|  | XP\_065053952\_4606-model12 | -7.53463 |
|  | XP\_065053952\_4606-model12.1 | -6.58694 |
| # cluster 13 - Size: 6 |  |  |
|  | XP\_065053952\_4606-model13 | -7.52279 |
|  | XP\_065053952\_4606-model13.1 | -7.45259 |
|  | XP\_065053952\_4606-model13.2 | -7.20947 |
|  | XP\_065053952\_4606-model13.3 | -7.20742 |
|  | XP\_065053952\_4606-model13.4 | -7.12244 |
|  | XP\_065053952\_4606-model13.5 | -6.84895 |
| # cluster 14 - Size: 5 |  |  |
|  | XP\_065053952\_4606-model14 | -7.44453 |
|  | XP\_065053952\_4606-model14.1 | -7.37729 |
|  | XP\_065053952\_4606-model14.2 | -7.3193 |
|  | XP\_065053952\_4606-model14.3 | -7.2379 |
|  | XP\_065053952\_4606-model14.4 | -6.86507 |
| # cluster 15 - Size: 2 |  |  |
|  | XP\_065053952\_4606-model15 | -7.44154 |
|  | XP\_065053952\_4606-model15.1 | -6.63921 |
| # cluster 16 - Size: 5 |  |  |
|  | XP\_065053952\_4606-model16 | -7.43676 |
|  | XP\_065053952\_4606-model16.1 | -7.37942 |
|  | XP\_065053952\_4606-model16.2 | -7.26694 |
|  | XP\_065053952\_4606-model16.3 | -6.88434 |
|  | XP\_065053952\_4606-model16.4 | -6.51283 |
| # cluster 17 - Size: 1 |  |  |
|  | XP\_065053952\_4606-model17 | -7.4258 |
| # cluster 18 - Size: 3 |  |  |
|  | XP\_065053952\_4606-model18 | -7.41647 |
|  | XP\_065053952\_4606-model18.1 | -7.13152 |
|  | XP\_065053952\_4606-model18.2 | -6.64459 |
| # cluster 19 - Size: 1 |  |  |
|  | XP\_065053952\_4606-model19 | -7.39422 |
| # cluster 20 - Size: 2 |  |  |
|  | XP\_065053952\_4606-model20 | -7.37377 |
|  | XP\_065053952\_4606-model20.1 | -6.70512 |
| # cluster 21 - Size: 2 |  |  |
|  | XP\_065053952\_4606-model21 | -7.30027 |
|  | XP\_065053952\_4606-model21.1 | -7.01433 |
| # cluster 22 - Size: 2 |  |  |
|  | XP\_065053952\_4606-model22 | -7.27645 |
|  | XP\_065053952\_4606-model22.1 | -7.05336 |
| # cluster 23 - Size: 4 |  |  |
|  | XP\_065053952\_4606-model23 | -7.23027 |
|  | XP\_065053952\_4606-model23.1 | -7.12291 |
|  | XP\_065053952\_4606-model23.2 | -6.86743 |
|  | XP\_065053952\_4606-model23.3 | -6.73221 |
| # cluster 24 - Size: 4 |  |  |
|  | XP\_065053952\_4606-model24 | -7.08164 |
|  | XP\_065053952\_4606-model24.1 | -7.02348 |
|  | XP\_065053952\_4606-model24.2 | -6.91683 |
|  | XP\_065053952\_4606-model24.3 | -6.73438 |
| # cluster 25 - Size: 2 |  |  |
|  | XP\_065053952\_4606-model25 | -7.07248 |
|  | XP\_065053952\_4606-model25.1 | -6.40365 |
| # cluster 26 - Size: 2 |  |  |
|  | XP\_065053952\_4606-model26 | -7.06581 |
|  | XP\_065053952\_4606-model26.1 | -7.00967 |
| # cluster 27 - Size: 1 |  |  |
|  | XP\_065053952\_4606-model27 | -7.00701 |
| # cluster 28 - Size: 3 |  |  |
|  | XP\_065053952\_4606-model28 | -6.99937 |
|  | XP\_065053952\_4606-model28.1 | -6.93925 |
|  | XP\_065053952\_4606-model28.2 | -6.73989 |
| # cluster 29 - Size: 1 |  |  |
|  | XP\_065053952\_4606-model29 | -6.96765 |
| # cluster 30 - Size: 2 |  |  |
|  | XP\_065053952\_4606-model30 | -6.90593 |
|  | XP\_065053952\_4606-model30.1 | -6.8813 |
| # cluster 31 - Size: 1 |  |  |
|  | XP\_065053952\_4606-model31 | -6.84848 |
| # cluster 32 - Size: 3 |  |  |
|  | XP\_065053952\_4606-model32 | -6.84791 |
|  | XP\_065053952\_4606-model32.1 | -6.78952 |
|  | XP\_065053952\_4606-model32.2 | -6.77771 |
| # cluster 33 - Size: 1 |  |  |
|  | XP\_065053952\_4606-model33 | -6.79967 |
| # cluster 34 - Size: 3 |  |  |
|  | XP\_065053952\_4606-model34 | -6.73299 |
|  | XP\_065053952\_4606-model34.1 | -6.60339 |
|  | XP\_065053952\_4606-model34.2 | -6.58683 |
| # cluster 35 - Size: 1 |  |  |
|  | XP\_065053952\_4606-model35 | -6.69711 |
| # cluster 36 - Size: 1 |  |  |
|  | XP\_065053952\_4606-model36 | -6.45072 |
|  |  |  |
